# Interaction between memories in an abstract mathematical model based on the Hebbian cell assembly hypothesis

**DOI:** 10.1186/1471-2202-16-S1-P253

**Published:** 2015-12-18

**Authors:** Juliane Herpich, Florentin Wörgötter, Christian Tetzlaff

**Affiliations:** 1 Third Physical Institute - Biophysics, Georg-August-University, Göttingen, Germany; 2 Bernstein Center for Computational Neuroscience, Göttingen, Germany

## 

Learning and memory are essential properties of adaptive neural circuits. Thereby, the general hypothesis (Synaptic-Plasticity-and-Memory hypothesis; [1,2]) is that the adaptive process of synaptic plasticity induces changes at the synapses connecting the neurons. These changes lead to the formation of strongly interconnected subgroups of neurons, so-called cell assemblies [3]. It has been suggested that such cell assemblies represent the learned memory items. However, as known from everyday life, after learning, humans and animals show the remarkable ability to connect, generalize, and discriminate old and new memories. How these memory interactions are realized on a neuronal level based on the idea of cell assemblies is still unknown.

In this work, we use a network model dependent on the interaction between synaptic plasticity and synaptic scaling [4,5]. Amongst others, this interaction yields the formation of cell assemblies showing dynamics comparable to human memories [6]. For simplicity, here, we further abstract this complex network model by the methods of mean-field theory. Thereby, the dynamics of each cell assembly in the network are reduced to two differential equations; one for the average neuronal activity and one for the average synaptic weight. Given this simplified model, we show that, in contrast to loosely connected groups of neurons, the formation of cell assemblies lets the activity of the neuronal population follow a hysteresis which creates complex network dynamics, which depend on the initial conditions. Given this hysteresis, in the next step, we connect two such assemblies with each other by plastic connections also adapted by the interaction between synaptic plasticity and scaling. Amongst others, we analyzed under which circumstances the dynamics of this two-cell-assembly system are comparable to the above mentioned dynamics of human and animal memories and how different parameters of the system (e.g., cell assembly size) influence them.

In summary, this work is one of the first using a mathematical model to relate the dynamics of memories on a psychological scale to the hypothesized dynamics of cell assemblies on a neuronal scale.
